# Thrombospondin1 antagonist peptide treatment attenuates obesity-associated chronic inflammation and metabolic disorders in a diet-induced obese mouse model

**DOI:** 10.1038/s41598-023-47635-2

**Published:** 2023-11-18

**Authors:** Qi Zhou, Taesik Gwag, Shuxia Wang

**Affiliations:** 1 Department of Pharmacology and Nutritional Sciences, University of Kentucky, Wethington Bldg. Room 583, 900 S. Limestone Street, Lexington, KY 40536 USA; 2 Lexington VA Medical Center, Lexington, KY 40502 USA

**Keywords:** Diseases, Molecular medicine

## Abstract

Thrombospondin1 (TSP1) is a multifunctional matricellular protein. Previously, we demonstrated that TSP1 plays a pivotal role in obesity-related inflammation and insulin resistance (IR) by modulating macrophage accumulation and activation in adipose tissue. Moreover, in our in vitro studies, a CD36-derived peptide, functioning as a TSP1 antagonist, effectively inhibited TSP1-induced proinflammatory macrophage activation. However, whether this CD36 peptide can inhibit obesity-induced inflammation and IR in vivo is unknown and determined in this study in a high fat diet-induced obese mouse model (DIO). CD36 peptide or control peptide was intraperitoneally administered into the established obese mice triweekly for 6 weeks. We found that CD36 peptide treatment didn’t affect obesity or weight gain but significantly reduced proinflammatory cytokine production systemically and in visceral fat tissue. Adipose tissue exhibited fewer crown-like structures and reduced macrophage infiltration. CD36 peptide treatment also attenuated the proinflammatory phenotype of bone marrow derived macrophages from obese mice. Furthermore, CD36 peptide treatment improved glucose tolerance and insulin sensitivity, and mitigated obesity-related fatty liver disease and kidney damage. Collectively, this study suggests that the CD36 peptide, as a TSP1 antagonist, shows promise as a novel therapeutic approach for managing obesity-related metabolic disorders.

## Introduction

Obesity and its associated metabolic disorders are becoming a global health problem. Accumulating evidence have shown that chronic inflammation is an important feature of obesity that promotes the development of insulin resistance (IR) and metabolic disorders^1^. Increased accumulation and/or activation of adipose tissue macrophages (ATMs) has been recognized as one of the significant contributors to inflammation in obesity and a key mediator of IR^1–3^. Although anti-inflammation has been considered as a therapeutic strategy for obesity-related disorders for many years, this therapy has not been very successful. Effective anti-inflammatory agents are still in urgent need for obesity treatment.

Thrombospondin1 (TSP1), a 420–450 kDa homotrimer with individual subunits of approximately 145 kDa, is a multifunctional matricellular protein composed of several domains that can interact with different cell surface receptors (e.g. CD36, integrins, CD47 etc.)^4^. TSP1 involves in cardiovascular and renal diseases as well as inflammatory diseases^5–18^. Recently, accumulating evidence from our lab and others suggest that TSP1 plays an important role in obesity-associated chronic inflammation and metabolic disorders (e.g. IR, kidney dysfunction and fatty liver disease (NAFLD))^19–23^. TSP1 is highly expressed in visceral adipose tissue (AT) from obese and insulin resistant humans or obese rodents^19,24–26^. Expression of TSP1 in AT is positively associated with AT inflammation and IR in obese human subjects^19^. Moreover, by utilization of global as well as tissue specific TSP1 deficient mouse model, our previous studies revealed a novel role for TSP1 in stimulating macrophage recruitment and activation in AT that contributes to inflammation, IR and NAFLD resulting from high fat diet-induced obesity (DIO)^20,27^. Mechanistically this effect is through TSP1 interacting with its-receptor-CD36^28^.

As a receptor of TSP1, CD36 is a membrane glycoprotein existing on many cell types including adipocytes and macrophages^29^. CD36 has been shown to be a key player in lipid metabolism and recently in obesity- associated inflammation and IR^29–37^. CD36 interacts with type 1 repeats of TSP1, which elicits signaling to negatively regulate angiogenesis or platelet activation^38–40^. Our previous studies revealed novel effects of TSP1/CD36’s interaction on TSP1-mediated macrophage activation^28^. We found that TSP1 treatment stimulated TNF-α production in macrophages, which could be inhibited by a known peptide derived from CD36 protein (p93–110) (called CD36 peptide^41,42^) to specifically block TSP1 binding to CD36^28^. However, the in vivo contribution of TSP1/CD36 interaction to obesity-induced inflammation and IR is unknown. Therefore, in this study, we determined whether specific blocking of TSP1/CD36 interaction with a CD36 peptide can ameliorate obesity-associated inflammation and IR in a diet-induced obesity mouse model.

## Results

### CD36 peptide administration did not affect body weight, but significantly improved glucose and lipid homeostasis in mice with established obesity

**Figure 1:**
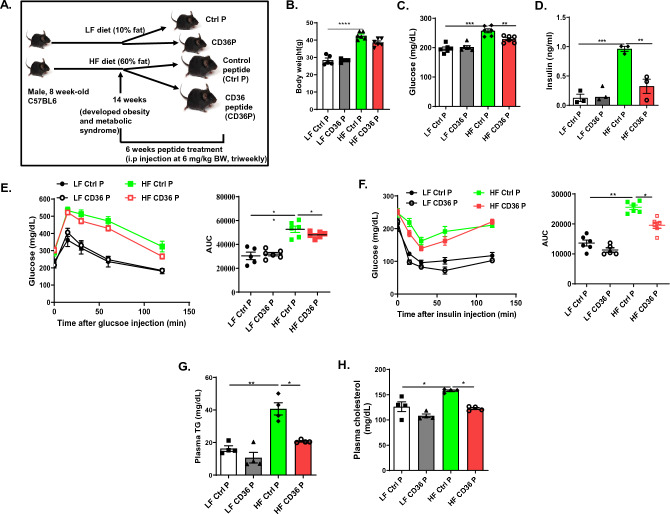
CD36 peptide administration did not affect body weight, but significantly improved glucose and lipid homeostasis in mice with established obesity. C57BL6 male mice (8 weeks-old) were fed with LF or HF diet for 14 weeks and then divided into two groups and received intraperitoneal injection of control peptide or CD36 peptide at 6 mg/kg triweekly for 6 weeks. (**A**) Study design; (**B**) Body weight; (**C**) Blood glucose levels; (**D**) Blood insulin levels; (**E**) Glucose tolerance test; (**F**) Insulin tolerance test; (**G**) Plasma triglyceride levels; and (**H**) Plasma total cholesterol levels. Data are represented as mean ± SEM (n = 5–6 mice/group). *P < 0.05, **P < 0.01, ***P < 0.001, and ****P < 0.0001.

To determine whether blocking the TSP1/CD36 interaction with a CD36 peptide can attenuate obesity-associated chronic inflammation and metabolic disorder in vivo, a diet-induced obesity mouse model (DIO) was utilized in this study. Control or CD36 peptide treatment was conducted in male C57BL6 mice after 14 weeks of high fat diet feeding when these mice developed obesity, chronic inflammation, and insulin resistance as compared to low fat feeding (LF) groups (Supplemental Fig. 1). Then, these mice were treated with control or CD36 peptide triweekly for 6 weeks (Fig. 1A). As shown in Fig. 1B, control or CD36 peptide treatment did not affect body weight changes in these mice. However, HF feeding induced glucose intolerance and reduced insulin sensitivity were significantly improved by CD36 peptide treatment as demonstrated by GTT and ITT assays as well as reduced fasting plasma glucose and insulin levels from obese mice (Fig. 1C–F). Obesity associated hypertriglyceridemia and hypercholesterolemia were also reduced by CD36 peptide treatment (Fig. 1G,H). This result suggests that the protective effect of CD36 peptide on glucose homeostasis or lipid homeostasis is independent of mice body weight changes, which is consistent with our previous findings showing that genetic deletion of TSP1 does not affect the development of diet-induced obesity^20^.

### CD36 peptide treatment suppressed systemic as well as adipose tissue inflammation in mice with established obesity

**Figure 2:**
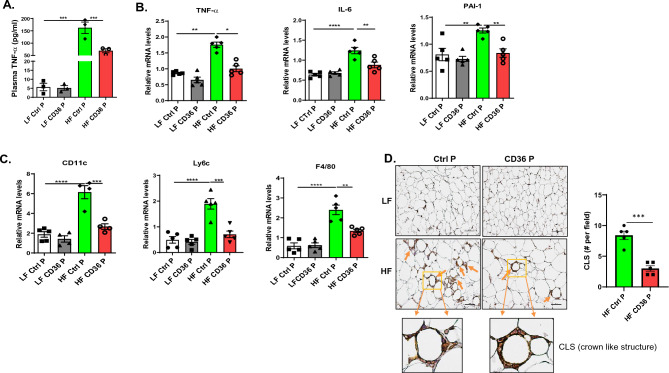
CD36 peptide treatment attenuated systemic as well as adipose tissue inflammation in HF diet fed mice. (**A**) Plasma TNF-α levels; (**B**) Expression of proinflammatory cytokines in epidydimal fat tissue by qPCR; (**C**) Expression of markers for monocyte/macrophages in epidydimal fat tissue by qPCR; (**D**) Representative adipose tissue immunohistochemical staining images for F4/80 (positive staining indicated by arrowhead, Scale bar = 100 µm). Enlarged picture showed CLS (crown like structure) and the CLS number was quantified. Data are represented as mean ± SEM (n = 5–6 mice/group). *P < 0.05, **P < 0.01, and ***P < 0.001.

**Figure 3:**
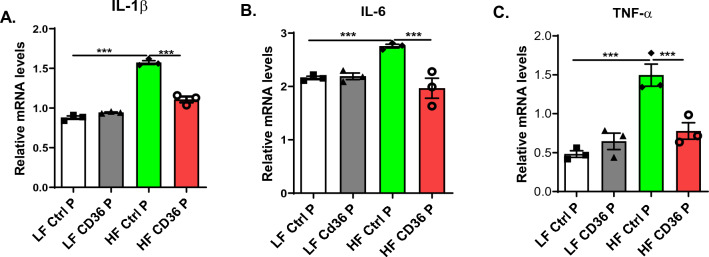
CD36 peptide treatment attenuated proinflammatory cytokine expression from bone marrow derived macrophages from obese mice. Bone marrow cells were isolated from femurs and tibias of male CD36 peptide or control peptide treated LF or HF fed C57 mice and differentiated into macrophages. Expression of proinflammatory genes (**A**) IL-1b, (**B**) IL-6 and (**C**) TNF-a were determined by qPCR. Data are represented as mean ± SEM (n = 3 experiments). *P < 0.05, **P < 0.01, and ***P < 0.001.

The mechanisms by which CD36 peptide treatment improved obesity-associated glucose homeostasis was determined. Accumulating evidence suggest that increased accumulation of adipose tissue macrophages (ATMs) is a significant contributor to obesity-induced chronic inflammation and a key mediator of insulin resistance^1–3^. Therefore, we determined the effect of CD36 peptide administration on ATM accumulation and adipose tissue inflammation, as well as systemic inflammation in obese mice. As shown in Fig. 2A**,** plasma TNF-alpha level was significantly increased in HF fed mice, which was attenuated by the CD36 peptide treatment. Similarly, increased expression of proinflammatory cytokines (e.g. TNF-alpha, PAI-1 and IL-6) or monocyte/macrophage markers (e.g. ly6c or F4/80) in adipose tissue from HF fed mice was suppressed by CD36 peptide treatment (Fig. 2B,C). Moreover, results from the immunohistochemical staining of epididymal fat tissue showed that HF feeding significantly increased F4/80 positive macrophage accumulation and crown like structure (CLS) in control peptide treated HF-fed mice, but reduced by CD36 peptide treatment (Fig. 2D). Negative control for IHC was shown in Supplemental Fig. 2. Interestingly, CD36 peptide treatment also attenuated the proinflammatory phenotype of bone marrow derived macrophages from obese mice by showing the reduced expression of TNF-alpha, IL-6 and IL-1 beta levels (Fig. 3). Together, the data indicate that CD36 peptide treatment significantly decreased macrophage accumulation and activation in adipose tissue as well as in bone marrow, leading to reduced systemic and local adipose tissue inflammation in HF fed obese mice.

### CD36 peptide treatment alleviated obesity-associated kidney damage

**Figure 4:**
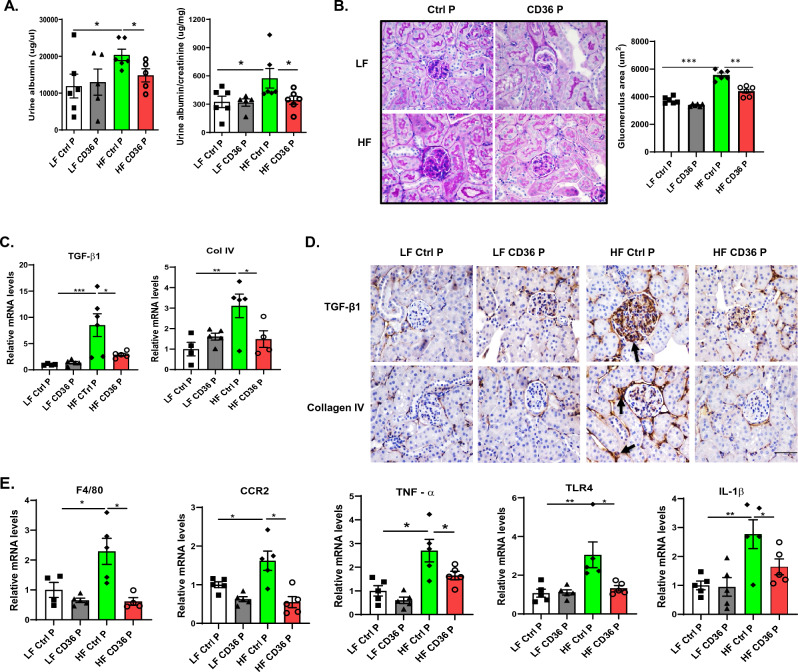
CD36 peptide treatment alleviated obesity-associated kidney damage. (**A**) At the end of study, 24 h urine was collected. Urine albumin level was measured and normalized to creatinine levels. (**B**) Representative images of kidney PAS (Periodic Acid Schiff) and quantification of glomeruli area from 4 groups of mice. (**C**) Expression of fibrosis related genes in kidney by qPCR. (**D**) Representative kidney immunohistochemical staining images for TGF-β1 and Collagen IV (positive staining shown as brown and indicated by arrow head, Scale bar = 100 µm). (**E**) Expression of proinflammatory genes in kidney by qPCR. Data are represented as mean ± SEM (n = 5–6 mice/group). *P < 0.05, **P < 0.01, and ***P < 0.001.

In addition to induce insulin resistance and the development of diabetes, obesity is also a major risk factor for organ dysfunction such as kidney and liver damages. Previously, we demonstrated that TSP1 is an important mediator of obesity induced kidney dysfunction partially through interaction with CD36^43–45^. Therefore, we first determined whether CD36 peptide treatment could attenuate obesity-associated kidney structural or functional changes. As anticipated, HF fed induced albuminuria, renal hypertrophy (by PAS staining and semi-quantification), and renal fibrosis (by qPCR analysis and IHC staining of TGF-beta 1 and Collagen IV) was attenuated by CD36 peptide treatment (Fig. 4A–D). This was accompanied by reduced expression of macrophages marker (F4/80) and proinflammatory cytokines (e.g. TNF-a, IL-1b, IL-6, CCR2, TLR4) in CD36 peptide treated mice (Fig. 4E). Triglyceride level in the kidney had a trend of decrease in CD36 peptide treated HF-fed mice as compared to control peptide treatment (data not shown). Collectively, these data show that blocking TSP1/CD36 interaction by CD36 peptide alleviates obesity-associated kidney inflammation and improves kidney function.

### CD36 peptide treatment attenuated obesity-associated liver damage

**Figure 5:**
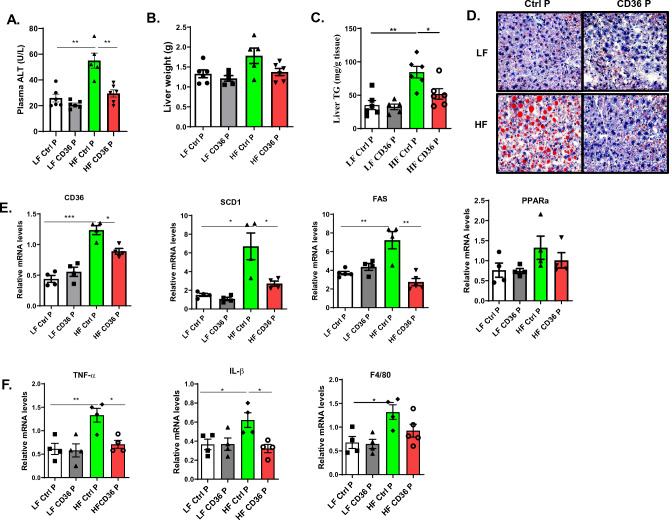
CD36 peptide treatment attenuated obesity-associated liver damage. (**A**) Plasma ALT levels, (**B**) Live weight, (**C**) Liver triglyceride levels, (**D**) representative liver Oil Red O staining images, (**E**) Expression of lipid metabolism related genes in liver by qPCR, (**F**) Expression of inflammatory cytokines in liver by qPCR. Data are represented as mean ± SEM (n = 4–6 mice/group). *P < 0.05, and **P < 0.01.

In addition to the protective effect on kidney function, we also determined the effect of CD36 peptide treatment on obesity-associated non-alcoholic fatty liver disease (NAFLD). Obesity is associated with the increased development of NAFLD. As expected, 20 weeks of 60% HF diet fed mice had increased plasma ALT and AST levels and developed simple steatosis demonstrated by Oil Red O staining of liver sections and analyzing liver triglyceride levels. This obesity-induced liver damage was attenuated by the CD36 peptide treatment showing reduced plasma ALT levels and liver fat accumulation (Fig. 5A–D). This was associated with reduced expression of genes related to lipid uptake or synthesis including SCD1, PPARα, CD36, and FAS expression (Fig. 5E). Additionally, expression of proinflammatory cytokines such as TNF-alpha and IL-1beta levels were reduced in CD36 peptide treated HF diet fed mice (Fig. 5F). These data indicate that CD36 peptide treatment attenuated obesity-associated liver damage.

## Discussion

In this study, the therapeutic potential of TSP1 antagonist peptide (CD36 peptide) in obesity-associated chronic inflammation and metabolic dysfunction was tested in HF diet-induced obese mouse model. We demonstrated that six weeks of CD36 peptide treatment attenuated adipose tissue as well as systemic inflammation in obese mice, leading to improved glucose homeostasis and attenuated liver and kidney damage. Importantly, this protective effect was independent of changes in body weight, suggesting that CD36 peptide, acting as an antagonist for TSP1, may offer a new therapeutic option for managing obesity-associated metabolic dysfunction.

In obesity, TSP1 is upregulated in adipose tissue and positively associated with obesity induced AT inflammation and insulin resistance^19^. Moreover, using a TSP1 deficient mouse model, we revealed a novel role for TSP1 in stimulating macrophage recruitment and activation in AT, contributing to obesity-associated metabolic disorders^20,27^. As expected, in the current study, CD36 peptide treatment did not affect obesity induced TSP1 expression in AT (Supplemental Fig. 3) but antagonized TSP1’s functions and reduced macrophage accumulation and proinflammatory activation in AT, resulting in reduced AT inflammation. In addition to directly regulating macrophage activation, other effects mediated by the TSP1/CD36 interaction may also contribute to obesity-induced inflammation. For example, blockade of TSP1/CD36 interaction by CD36 peptide stimulates angiogenesis^39^. Improved blood supply may lead to healthier adipose tissue expansion and/or remodeling under HF feeding conditions^46^. Blockade of TSP1/CD36 interaction may promote fatty acid uptake^38^, reducing free fatty acid levels from the circulation. All of these effects may contribute to the reduction in obesity-induced inflammation mediated by CD36 peptide treatment. Furthermore, TSP1 is a major regulator for latent TGF-β activation in vitro as well as in vivo^47–51^. TSP1/CD36 interaction has been shown to activate latent TGF-β in rat alveolar macrophage^52^. Studies have demonstrated that increased TGF-β activity and its downstream target PAI-1 are associated with obesity, inflammation and insulin resistance^53–55^. We found that TGF-β downstream molecular-PAI-1 levels in adipose tissue were significantly decreased in CD36 peptide treated obese mice (Fig. 2B), suggesting that reduced TSP1 dependent TGF-β activity may also contribute to the reduced systemic and local inflammation and improved insulin sensitivity observed in CD36 peptide treated obese mice. These possible mechanism warrants further investigation.

In addition to attenuation of obesity-induced inflammation and insulin resistence in obese mice, CD36 peptide treatment alleviated obesity-associated kidney or liver damage. Previoulsy, we found that renal TSP1 was upregulated in HF diet-induced obese mice, which was associated with the development of albuminuria, increased kidney macrophage infiltration, augmented kidney inflammation, and increased fibrosis^43^. Knock out of TSP1 or CD36 improved obesity-associated kidney damage^43–45^. Consistently, in this study, CD36 petide mediated TSP1 antagonization attenuated obesity-induced kidney damage. This is partially through downregulation of kidney macrophage infiltration and proinflammatory activation. Other mechanisms may also involve. For instance, CD36 peptide treatment might block TSP1’s effect on podocyte function^44^. Our previous studies demonstreated that free fatty acid (FFA) stimulated TSP1 expression in podocytes, which bound to CD36 to induce podocyte undergo apoptosis^44^. Therefore, CD36 peptide treatment may attenuate obesity/FFA induced podocyte apoptosis, reduce albuminuria and improve kidney function.

Notably, in the current study, we revealed that CD36 peptide treatment effectively mitigated obesity-associated non-alcoholic fatty liver disease (NAFLD), as evidenced by reduced plasma ALT and AST levels, as well as decreased liver fat accumulation. This beneficial effect may operate through a TSP1-independent mechanism. Interestingly, our previous research, which explored the impact of TSP1 deficiency on NAFLD using both global and myeloid-specific TSP1 null mice, did not reveal any significant influence on fatty liver development^20,23^. This suggests that the improvements observed in our current study by CD36 peptide treatment may be attributable to the enhanced insulin sensitivity or a reduction in local fatty acid uptake and hepatic lipogenesis, which is supported by the reduction in the expression of genes related to liver lipid metabolism (Fig. 5).

In summary, our studies demonstrated that CD36 peptide treatment reduced obesity associated chronic inflammation, leading to improved glucose homeostasis and reduced kidney and liver damage in obese mice. The results for this study suggest that CD36 peptide, as a TSP1 antagonist, may serve as a new therapeutic option for obesity-associated comorbidities.

## Methods

### Animal experiments

All the experiments involving mice conformed to the National Institutes of Health Guide for the Care and Use of Laboratory Animals and were approved by the University of Kentucky Institutional Animal Care and Use Committee. This study is reported in accordance with ARRIVE guidelines. All animals were housed in a pathogen-free environment with a light–dark cycle. Male eight-week-old C57BL/6 wild type (WT) mice (The Jackson Laboratory; Bar Harbor, ME) were fed with high fat diet (HFD) (60% kcal from fat, D12492, Research Diets, Inc, NJ) for 14 weeks to induce obesity (DIO), chronic inflammation and insulin resistance (IR). DIO mice were then divided into two groups and intraperitonially injected with control peptide (RFAYLRKNVTENDEQAVCD) or CD36 peptide (YRVRFLAKENVTQDAEDN) from American Peptide Company Inc. at 6 mg/kg body weight triweekly for 6 weeks. Body weight was measured weekly. At the end of the study, mice were sacrificed. Blood was collected and adipose tissue depots and other organs were harvested for various analyses. Low fat (LF) diet (10% kcal form fat; D12450B; Research Diets, Inc, NJ) fed mice were also included in the study.

### Intraperitoneal glucose (GTT) and insulin (ITT) tolerance test

Glucose tolerance and insulin sensitivity tests were analyzed at the end of the study. Mice were fasted for 6 h prior to receiving intraperitoneal injections of glucose (1 g/kg body weight) or insulin (0.5 units/kg body weight; Novolin R, Novo Nordisk Inc.). Blood glucose concentrations were measured using a glucometer at 0, 15, 30, 60, and 120 min post-injection.

### Blood or plasma parameter analysis

Blood glucose levels were measured by using glucometer. Plasma insulin and TNFα levels were measured by using ELISA Kit (eBioscience, Waltham, MA). Plasma total cholesterol and triglyceride, and hepatic cholesterol and triglyceride concentrations were determined enzymatically with Wako kits (Richmond, USA).

### RNA isolation and qPCR analysis

Total RNA was isolated from mouse fat, liver, and kidney tissues using Trizol Reagent (Invitrogen Life Technologies; Carlsbad, CA). RNA was then reverse transcribed into cDNA using the High Capacity cDNA Reverse Transcription Kit (Invitrogen, Carlsbad, CA). Real-time quantitative PCR was conducted on a MyiQ Real-time PCR Thermal Cycler (Bio-Rad) with the SYBR Green PCR Master Kit (Qiagen, Valencia, CA). Relative mRNA expression levels were calculated using the MyiQ system software, as previously reported^20^, and normalized to 18sRNA levels. The primer sequences used in this study can be found in Table 1.

**Table 1:** Primer sequences for qPCR.

Gene	Primer sequence	Genes	Primer sequence
Mouse primers			
FAS	5′-TCCTGGAACGAGAACACGATCT-3′ 5′-GAGACGTGTCACTCCTGGACTTG-3′	SCD-1	5′-TTCTTGCGATACACTCTGGTGC-3′ 5′-CGGGATTGAATGTTCTTGTCGT-3′
IL6	5′-CTGCAAGAGACTTCCATCCAGTT-3′ 5′-GAAGTAGGGAAGGCCGTGG-3′	PAI1	5′-GCGTGTCAGCTCGTCTACAG-3′ 5′-GTACTGCGGATGCCATCTTT-3′
Ly6c	5′-GTGTGCTCATTCTTCTTG-3′ 5′-TGTTAGGATCCCTGATTG-3′	Collagen IV	5′-AGGGTTCCCAGGTTCTAA-3′ 5′-GCCCAACGTCACCTTTAT-3′
F4/80	5′-CTTTGGCTATGGGCTTCCAGTC-3′ 5′-GCAAGGAGGACAGAGTTTATCGTG-3′	CCR2	5′-AGAGAGCTGCAGCAAAAAGG-3′ 5′-GGAAAGAGGCAGTTGCAAAG-3′
CD11c	5′-CTGGATAGCCTTTCTTCTGCTG-3′ 5′-GCACACTGTGTCCGAACTC-3′	Tlr4	5′-AACCAGCTGTATTCCCTCAGCACT-3′ 5′-ACTGCTTCTGTTCCTTGACCCACT-3′
IL-1β	5′-TGGAGAGTGTGGATCCCAAGCAAT-3′ 5′-TGTCCTGACCACTGTTGTTTCCCA-3′	TNFα	5′-AGCCGATGGGTTGTACCT-3′ 5′-TGAGTTGGTCCCCCTTCT-3′
TGFβ	5′-ACAATTCCTGGCGTTACC-3′ 5′-GGCTGATCCCGTTGATTT-3′	PPARα	5′-CAGCCTCAGCCAAGTTGAAGT-3′ 5′-CGAACTTGACCAGCCACAAA-3′
CD36	5′-ACTGGTGGATGGTTTCCTAGCCTT-3′ 5′-TTTCTCGCCAACTCCCAGGTACAA-3′	18sRNA	5′-AGTCGGCATCGTTTATGGTC-3′ 5′-CGAAAGCATTTGCCAAGAAT-3′

### Immunohistochemical staining of fat and kidney tissues

Epididymal adipose tissue or kidney samples were fixed and embedded in paraffin. Paraffin-fixed tissues were then sectioned into 4 µm slices and mounted onto slides. The sections underwent de-paraffinization and rehydration through a series of graded ethanol/water mixtures. They were further pretreated by boiling in citrate buffer (pH 6.0), and endogenous peroxidase activity was blocked with 3% H_2_O_2_ for 30 min at room temperature (RT). For adipose tissue sections, a rat anti-mouse F4/80 antibody (AbD Serotec, Raleigh, NC) was used, while kidney sections were incubated with an anti-Collagen IV antibody (Cell Signaling) in blocking buffer for 1 h at room temperature. Negative control sections without primary antibody incubation were included. Afterward, the sections were washed and incubated with a biotinylated secondary antibody for 30 min. Finally, a peroxidase substrate, diaminobenzidine (Vector Lab), was applied and incubated for 30 min. The slides were rinsed, counterstained with hematoxylin, and then mounted. Images were captured using a Nikon Eclipse 55i microscope.

### Kidney function and histology analysis

At the end of study, mice were placed in metabolic cages to collect urine samples. The levels of urinary albumin and creatinine were assessed using mouse albumin ELISA and creatinine companion kits, respectively, following the manufacturer's instructions (Philadelphia, PA). Additionally, serum creatinine levels were measured using a kit provided by Biovision (Milpitas, CA).

For the histological analysis of the kidneys, the kidneys were harvested and fixed in a 10% neutral formalin solution. Subsequently, they were embedded in paraffin and sectioned into 4-μm-thick sections. After deparaffinization, the tissue sections were rehydrated and stained with periodic acid-Schiff (PAS) reagent obtained from Sigma. Based on the PAS staining, pathological changes in the glomeruli were examined under a light microscope. The analysis of glomerular area was conducted blindly using computer imaging software.

### Liver lipid analysis

For analysis of hepatic lipid, approximately 50 mg of liver was placed into 500 μl of chilled Krebs Ringer Phosphate buffer (118 mM NaCl, 5 mM KCl, 13.8 mM CaCl_2_, 1.2 mM MgSO_4_, 0.016% KH_2_PO_4_, 0.211% NaHCO_2_) and each sample was sonicated for ten times (30 s/time). In addition, hepatic lipid accumulation was also determined by staining frozen liver sections with Oil Red O as previously described^56^.

### Bone marrow derived cell isolation and differentiation into macrophages

At the end of study, bone-marrow derived cells were isolated from femurs and tibias of male CD36 peptide or control peptide treated LF or HF fed C57 mice as described previously^57^. For seven days, these cells were cultured in RPMI-1640 media containing 20% FBS, 25 ng/ml M-CSF (Sigma), and penicillin/streptomycin to allow proliferation and differentiation into mature macrophages. Total RNA was extracted from these cells and qPCR was performed for gene analyzation.

### Statistical analysis

Statistical analysis was conducted using Prism version 8.0.2 software (GraphPad Software, San Diego, CA). The data are expressed as the mean ± SEM (standard error of the mean). Individual pairwise comparisons were analyzed using a two-sample, two-tailed Student’s *t*-test, unless otherwise specified, with significance set at P < 0.05. In cases where multiple comparisons were necessary, a one-way analysis of variance (ANOVA) was employed, followed by Turkey’s multiple comparisons test. The specific N numbers for each analysis are provided in the figure legends.

### Supplementary Information

Supplementary Figures.

## Data Availability

All data generated or analyzed during this study are included in this published article and its supplementary information files.
