# Supplementary material for: Thrombospondin1 antagonist peptide treatment attenuates obesity-associated chronic inflammation and metabolic disorders in a diet-induced obese mouse model
Source: Sci Rep. 2023 Nov 18;13:20193. doi: 10.1038/s41598-023-47635-2 (PMC10657402; doi:10.1038/s41598-023-47635-2)

Thrombospondin1 antagonist peptide treatment attenuates obesity-associated chronic inflammation and metabolic disorders in a diet-induced obese mouse model

Qi Zhou, Taesik Gwag, and Shuxia Wang*

Department of Pharmacology and Nutritional Sciences, University of Kentucky, Lexington, KY 40536, and Lexington VA Medical Center, Lexington KY 40502.

Fig. S1: Fourteen weeks of high fat diet feeding induced obesity, inflammation, and insulin resistance in male C57BL6 mice

Male 6-week old C57BL6 mice were fed with LF or HF diet for fourteen weeks. (A). Body weight; (B). Plasma TNF-a levels; (C) Plasma glucose level; (D) Plasma Insulin level; (E) Glucose tolerance test (GTT) and (F) Insulin tolerance test (ITT) and area under the curve were analyzed. Data are represented as mean ± SE (n=14-16 mice/group). * $P<0.05$, ** $P<0.01$, *** $P<0.001$

Fig. S2: Representative negative control images for immunohistochemical staining of fat tissue and liver

Immunohistochemical staining was performed in epidydimal fat tissue (A) or liver (B) in the absence of primary antibodies (F/80, Collagen IV, TGF-beta 1) to serve as negative controls for Figure 2D and Figure 4D IHC. The representative staining images are shown.

Fig. S3. Obesity upregulated tissue TSP1 expression, which was not affected by CD36 peptide treatment

Expression of TSP1 in epidydimal fat, liver, kidney and bone-marrow derived macrophages from different groups of mice was determined by qPCR. Data are represented as mean ± SEM (n=3-4 mice/group). *$P<0.05$, ** $P<0.01$, and *** $P<0.001$

Supplemental Figure 1

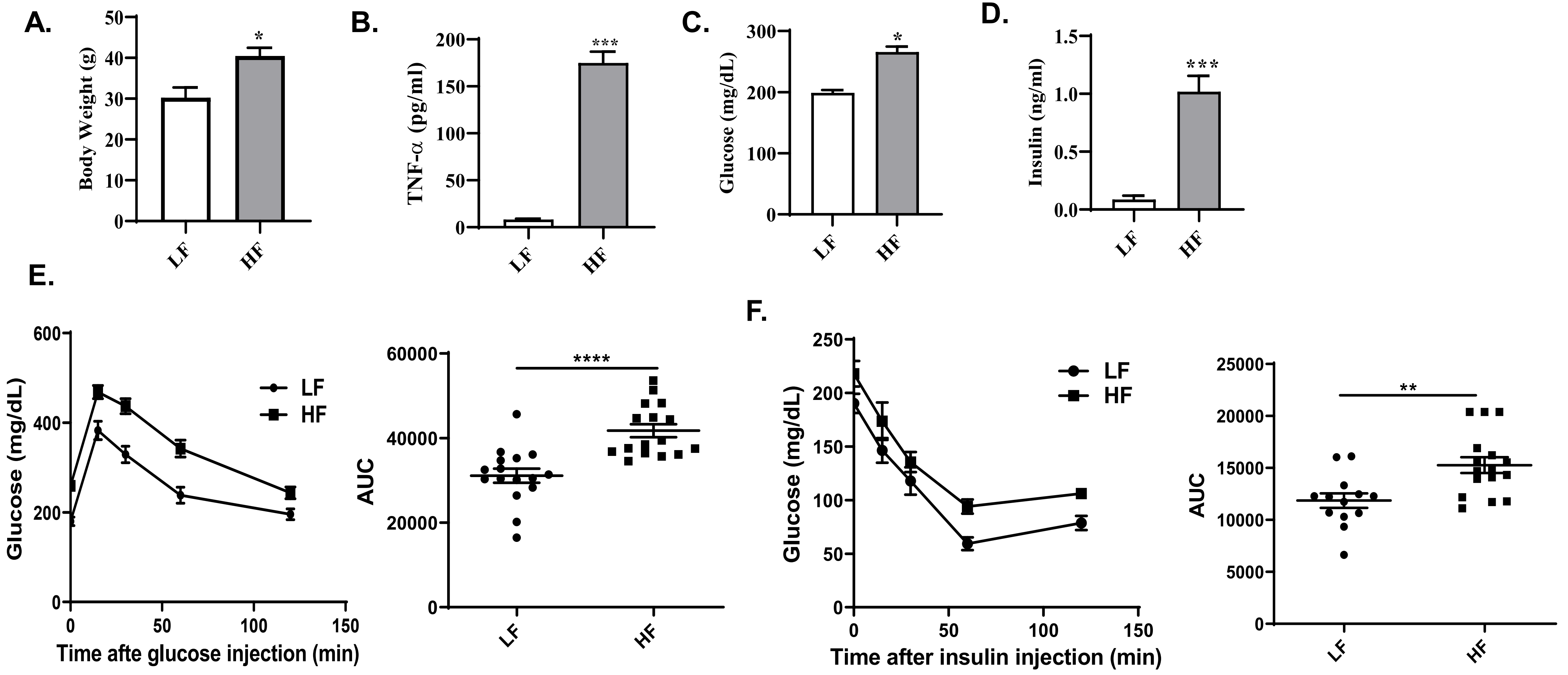

Supplemental Figure 2

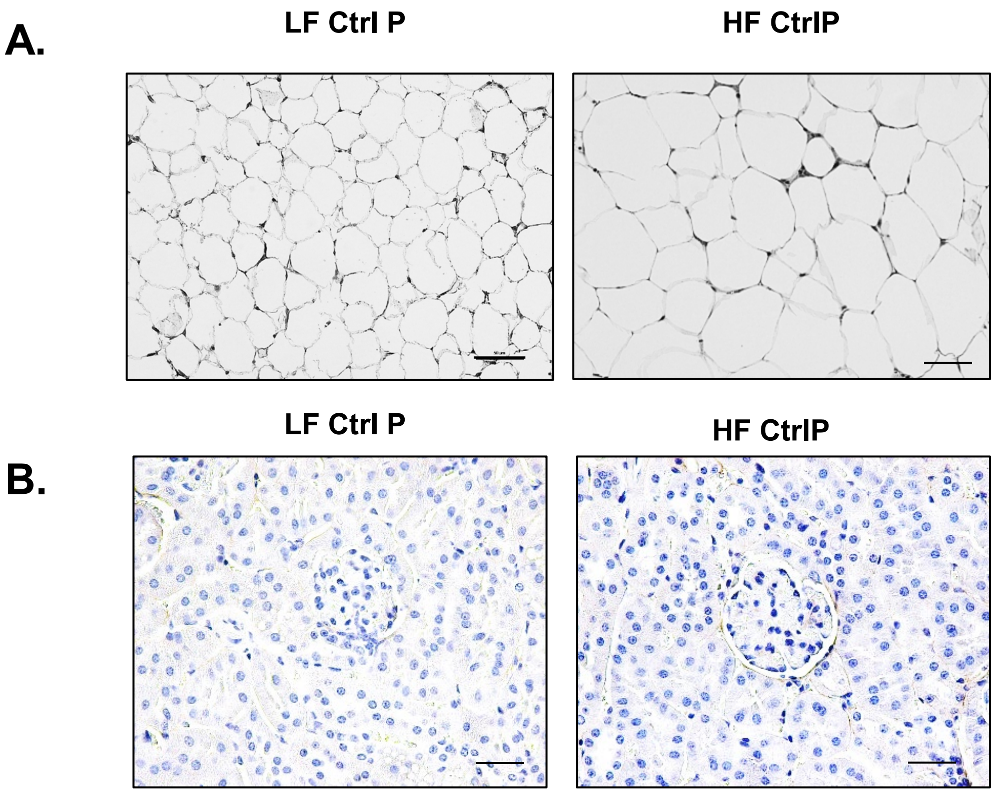

Supplemental Figure 3

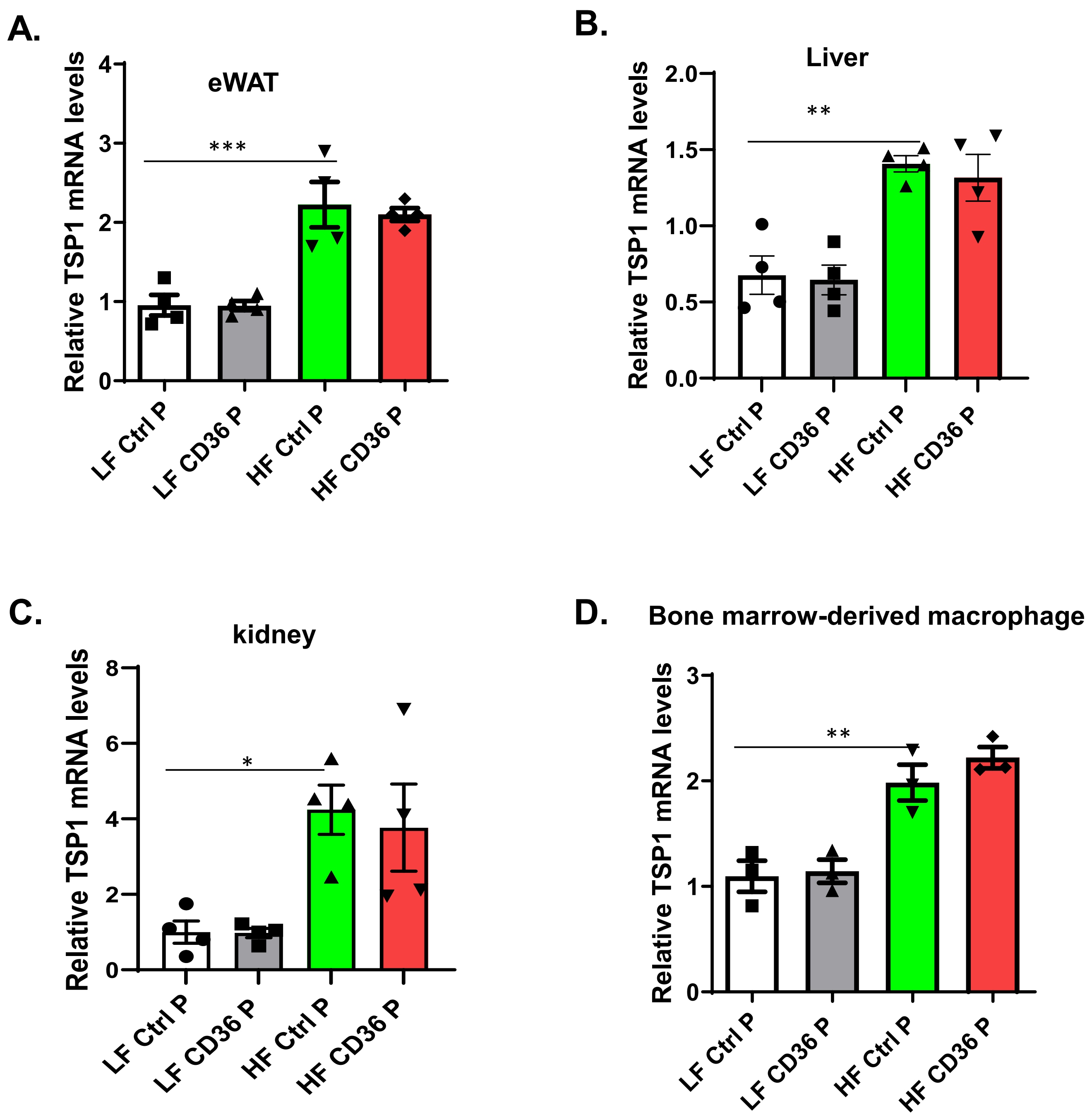

Supplement: Supplementary file 1 — Supplementary Figures. [file 41598_2023_47635_MOESM1_ESM.pdf]
